# Improving Assignments for Therapeutic and Prophylactic Treatment Within TB Households. A Potential for Immuno-Diagnosis?

**DOI:** 10.3389/fimmu.2022.801616

**Published:** 2022-03-17

**Authors:** Dhanasekaran Sivakumaran, Synne Jenum, Christian Ritz, Mario Vaz, Timothy Mark Doherty, Harleen M. S. Grewal

**Affiliations:** ^1^ Department of Clinical Science, Bergen Integrated Diagnostic Stewardship Cluster, Faculty of Medicine, University of Bergen, Bergen, Norway; ^2^ Department of Microbiology, Haukeland University Hospital, University of Bergen, Bergen, Norway; ^3^ Department of Infectious Diseases, Oslo University Hospital, Oslo, Norway; ^4^ National Institute of Public Health, University of Southern Denmark, Copenhagen, Denmark; ^5^ Department of Physiology, St. John’s Medical College, Bangalore, India; ^6^ Division of Health and Humanities, St. John’s Research Institute, Bangalore, India; ^7^ GlaxoSmithKline Vaccines, Wavre, Belgium

**Keywords:** *Mtb* infection, protein signature, active TB, preventive therapy, soluble protein markers, cytokine and chemokines

## Abstract

Delays in diagnosis and treatment of pulmonary tuberculosis (TB) can lead to more severe disease and increased transmission. Contact investigation among household contacts (HHCs) of TB patients is crucial to ensure optimal outcomes. In the context of a prospective cohort study in Palamaner, Southern India, this study attempted to assess the potential of 27 different soluble immune markers to accurately assign HHCs for appropriate treatment. A multiplex bead assay was applied on QuantiFERON (QFT)-nil supernatants collected from 89 HHCs grouped by longitudinal QFT status; *M. tuberculosis* (*Mtb*) infected (QFT positive at baseline and follow-up, n = 30), recent QFT converters (QFT-negative at baseline, n = 27) and converted to QFT-positivity within 6 months of exposure (at follow-up, n = 24) and QFT consistent negatives (n = 32). The 29 TB index cases represented Active TB. Active TB cases and HHCs with *Mtb* infection produced significantly different levels of both pro-inflammatory (IFNγ, IL17, IL8, IP10, MIP-1α, MIP1β, and VEGF) and anti-inflammatory (IL9 and IL1RA) cytokines. We identified a 4-protein signature (bFGF, IFNγ, IL9, and IP10) that correctly classified HHCs with *Mtb* infection vs. Active TB with a specificity of 92.6%, suggesting that this 4-protein signature has the potential to assign HHCs for either full-length TB treatment or preventive TB treatment. We further identified a 4-protein signature (bFGF, GCSF, IFNγ, and IL1RA) that differentiated HHCs with *Mtb* infection from QFT consistent negatives with a specificity of 62.5%, but not satisfactory to safely assign HHCs to no preventive TB treatment. QFT conversion, reflecting new *Mtb* infection, induced an elevated median concentration in nearly two-thirds (19/27) of the analyzed soluble markers compared to the levels measured at baseline. Validation in other studies is warranted in order to establish the potential of the immune biosignatures for optimized TB case detection and assignment to therapeutic and preventive treatment of *Mtb* infected individuals.

## Introduction

Even before the impact of the COVID-19 pandemic on health system capacity, the decline in TB incidence by 2019 was already too slow to reach the first milestone of the End TB Strategy, with still an estimated number of 10.0 million new TB cases annually (1). The COVID-19 pandemic has impacted everyday life, damaged economies, and overwhelmed health care systems (2, 3). The interruption of health services to a point where identification of new TB cases and treatment initiation decreased by 25–50% over 3 months (between April and June, 2020) may upsurge TB deaths by 0.2–0.4 million globally in 2020 alone (1). Notably, early identification and treatment is a priority of the End TB Strategy to minimize transmission mandatory to reach the goal of eliminating the TB epidemic (4, 5).

The risk of TB progression is highest in newly exposed subjects; thus, active case finding in the households of newly diagnosed TB index patients is considered an effective strategy (6). The purpose of contact investigations is to identify subjects with increased risk of TB progression in order to offer preventive TB treatment (7). The WHO recommendations for preventive TB treatment depend on the national health resource setting and the epidemiological context. In low- and middle-income countries, preventive TB treatment is recommended for high-risk subjects such as children aged below 5 years and people living with HIV or other immunosuppressant conditions. Active TB must be ruled out by assuring the absence of the hallmark TB symptoms; cough, fever, weight loss and night sweats (8). In order to achieve early case identification and avoid TB progression with the risk of subsequent transmission, TB control programs could profit from more ambitious goals: To identify all household contacts with active/subclinical TB or *M. tuberculosis* (*Mtb*) infection at risk of TB progression, and, dependent on the condition, assign appropriate treatment. For active/subclinical TB (drug-sensitive *Mtb* isolates) this would be a full 6-month course (2-month intensive phase; isoniazid, rifampicin, pyrazinamide, and ethambutol) followed by a 4-month continuation phase; isoniazid, rifampicin), and for *Mtb* infected a prophylactic course of 3 months (isoniazid, rifampicin/rifapentine) either once-weekly or daily, or 9 months (isoniazid) either twice-weekly or daily (9). Accurate assignment of patients is vital to avoid under- and over-treatment, risking either relapse and/or *Mtb* resistance in the case of undertreating, or side-effects without gain in the case of overtreating (10).

It has repeatedly been shown that despite heavy and prolonged *Mtb* exposure, a certain proportion of subjects does not develop detectable T cell responses (for e.g., they remain QFT negative through repeated testing). These subjects have the lowest risk of TB progression, suggesting that they possess effective natural TB immunity, likely mediated by innate immune mechanisms (11, 12). No proxy marker or biosignature for this kind of natural protective immunity has been established, but finding such a biomarker or biosignature is considered the holy grail within TB immunology (13): Immunobiomarkers in TB may be useful to understand the role of host responses in natural protection or vulnerability towards *Mtb* infection, but also hold a potential for new diagnostic tools and proxy-readouts for vaccine and/or treatment efficacy (14).

In the setting of a longitudinal study of index TB cases and their household contacts, the Household Contact Cohort (HHC) study, conducted in Palamaner, Southern India, in the period 2010–2012, aimed to explore the potential of 27 soluble immune markers detected in QuantiFERON (QFT) nil tube supernatants to accurately assign TB-exposed subjects within a household to either i) standard TB treatment (concomitant active and/or subclinical TB cases) or ii) preventive treatment (*Mtb* infected with risk of TB progression) (15) or eventually iii) no treatment in subjects that likely possess natural immunity. Furthermore, we aimed to explore if the selected markers could provide some insight into natural TB immunity.

## Methods and Materials

### Ethical Consideration

Ethical approval for this study was obtained from the Institutional Ethical Review Board (IERB) of St. John’s Medical College, Bangalore (IERB/1/527/08). The material transfer agreement between St. John’s Medical College, Bangalore, and the University of Bergen, Norway, was obtained from the Department of Biotechnology, Government of India (No. BT/Med.II/Adv (SS)/Misc./02/2012). Ethical approval was also obtained from Western Norway’s Regional Committee for Medical and Health Research Ethics (Ref no: **2018/1614 D**).

### Study Population

The present study is a cross-sectional study nested within a prospective cohort study of adult PTB index cases and their household contacts and conducted at our study site at the Emmaus Swiss Leprosy Project and Referral Hospital, Palamaner and Kuppam Taluks, Chittoor district, Andhra Pradesh, India (3.200°N, 72.7500°E, altitude 683 m) between September 2010 and April 2012. In total, 176 pulmonary TB (PTB) index cases were identified at the microscopy centers of the Revised National Tuberculosis Control Program (RNTCP) run by the Government of India and 164 recruited after written informed consent. In the context of our study, TB was confirmed in 150 cases by the presence of *Mtb* in sputum smear and/or culture. Positive cultures were confirmed by using the GenoType MTBC kit (Hain Life science GmBH, Nehren, Germany). Direct PCR by COBAS Taqman MTB test (Roche Diagnostics Ltd, Rotkreuz, Switzerland) was undertaken on culture-negative specimen for patients with chest X-ray (CXR) finding suggestive of TB. Recruited PTB index cases were treated with standard anti-TB treatment (ATT) and followed until completion of the 6-month treatment course. A total of 525 household contacts of the 176 PTB index cases were recruited. Household contacts who were not part of the 150 *Mtb* culture-positive index TB cases were also included for the investigation. Written informed consent was provided by all adults. For children ≤7 years, parents/guardians provided written informed consent, and for participants >7 years, an additional written assent was obtained. The intended follow-up for all household contacts was one year.

### Clinical Assessments and Sampling

*Baseline assessments of PTB index cases and household contacts:* Medical history (namely, BCG vaccination status, history of TB exposure, prior TB/ATT, socio-economic status, and risk factors such as diabetes, smoking, and alcohol consumption), socio-demographic, anthropometric, and clinical data were recorded. At baseline, a tuberculin skin test (TST) was performed by a trained nurse (2 TU/0.1 ml tuberculin; Span Diagnostics, Surat, India) and read after 48–72 h; an induration >10 mm was defined as positive. Blood 3 ml was collected for the QuantiFERON-TB Gold In-tube (QFT-GIT) assay and the supernatants (plasma) from the Nil, TB antigen (ag) and Mitogen tubes were collected in two microfuge tubes and stored at −80°C. One microfuge tube was used for the QFT-GIT assay and the other tube was stored for Bio-Plex assay. Three independent radiologists interpreted the CXR (anteroposterior view) at baseline, and agreement by at least two was required for the assignment abnormal TB chest X-ray. CXR was again done at the end of the study closure for all participants. Since the prevalence of HIV in India is low [<0.5%] (16), after pre-test counseling, only participants who volunteered for HIV testing were tested. Agreement for HIV testing was not a criterion for inclusion.

### Sample Selection and Definition of Groups

PTB index cases were selected to represent Active TB disease (Group 1) as concomitant cases in the household were referred to the RNTCP and excluded from the HHC. Stored blood samples available from 109 of 150 *Mtb* culture-positive participants, and only 48 of 109 blood samples were obtained before ATT initiation for QFT, of which only 39 index TB cases had either previously diagnosed TB >3 years or no prior TB reported. These 39 samples were considered to include in this study. However, QFT Nil tube samples were available for 29 of 39 PTB index cases and constituted Group 1, Active TB disease.

Household contacts being QFT positive at baseline and at repeated testing at 2-, 6-, and 12-month follow-up and did not develop TB disease, were assigned as QFT consistent positive *Mtb* infected (Group 2) and were considered representative of historical and/or likely recent *Mtb* exposure.

Household contacts, who were QFT negative at baseline but converted to QFT positivity at 2- or 6-month follow-up and did not develop TB disease, were assigned to recent QFT converters (Group 3) and were considered representative of recent *Mtb* exposure.

Household contacts that remained QFT negative throughout the 12-month follow-up and that did not develop TB were assigned to QFT consistent negatives (Group 4) and were considered representative of likely natural protective innate mechanisms towards TB.

Both *Mtb* infected (QFT consistent positive) and recent QFT converters (QFT positivity at 2- or 6-month follow-up) were considered representative of contacts that would profit from preventive treatment without risk of undertreating subclinical TB/active TB. Therefore, these two groups were merged and called Household contacts with *Mtb* infection.

**Figure 1:**
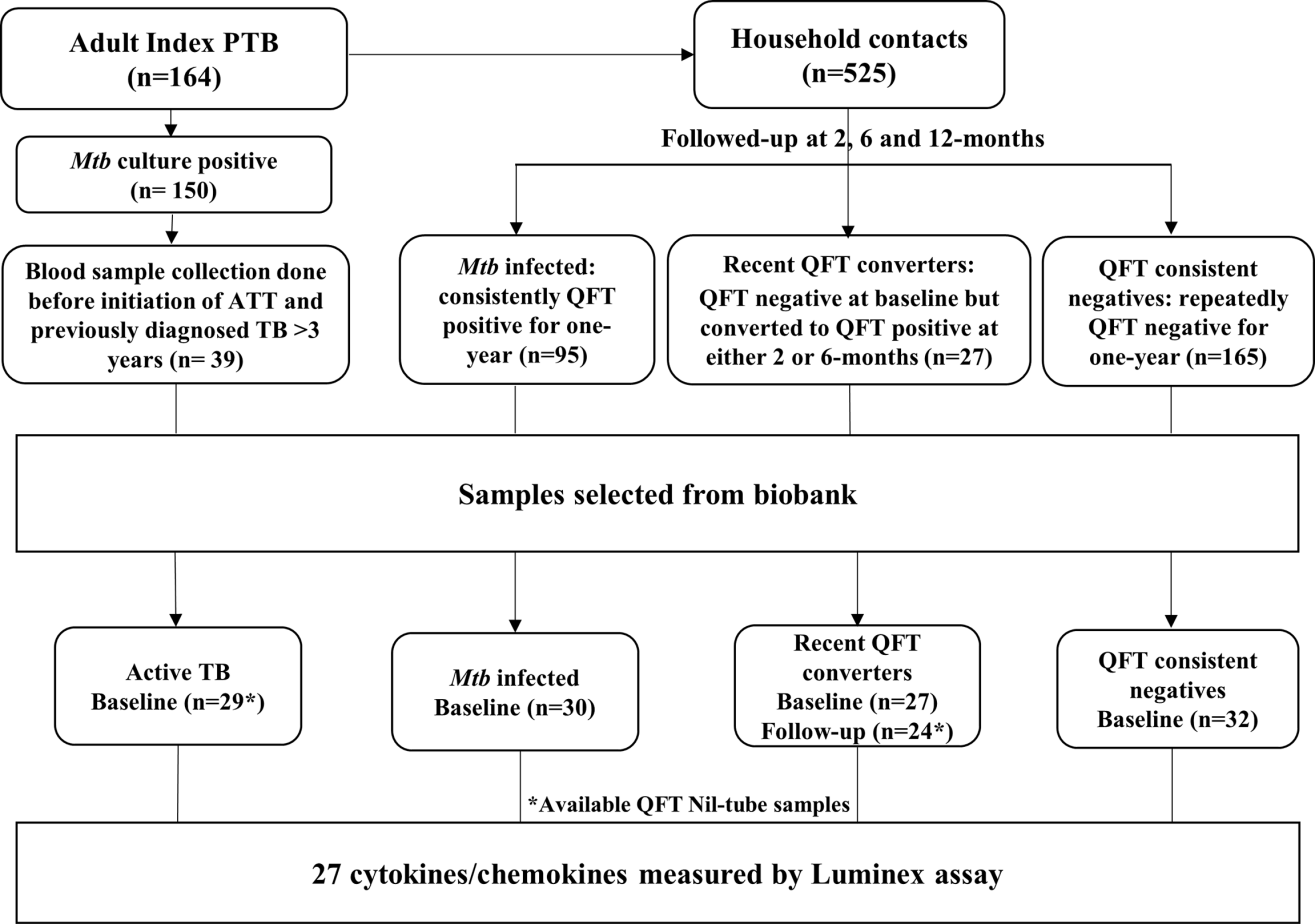
Study flow chart.

Sample selection is shown in **Figure 1**. For the purpose of this study, Active TB (n = 29), QFT consistent positive *Mtb* infected (n = 30; hereafter referred to as *Mtb* infected throughout this manuscript), recent QFT converters (n = 27 who were QFT-negative at baseline but who converted to QFT-positivity within 6 months of exposure; 14 converted at 2 months and 10 converted at 6 months and for 3 samples the follow-up Nil-ag tubes were not available) and QFT consistent negatives (n = 32). In total, 142 samples were included for the biomarker analysis.

### Multiplex Cytokine/Chemokine Assays

For the Bio-Plex Multiplex Immunoassays, we used the Nil tube supernatants from the QFT-GIT assay. This unstimulated control sample can be used as a substitute for separate serum stored samples (17). The selected 142 samples were tested using the ‘Human cytokines 27-plex’ kit (Bio-Rad Laboratories Inc., Hercules, CA, USA), according to the instructions of the manufacturer. The 27 biomarkers included are Interleukin (IL)-1β, IL-1 receptor antagonist (IL1-ra), IL-2, IL-4, IL-5, IL-6, IL-7, IL-8/CXCL8, IL-9, IL-10, IL-12 (p70), IL-13, IL-15, IL-17, eotaxin/CXCL11, basic fibroblast growth factor (bFGF), granulocyte-colony stimulating factor (G-CSF), granulocyte–macrophage colony-stimulating factor (GM-CSF), interferon (IFN)-γ, interferon-inducible protein (IP-10)/CXCL10, monocyte chemotactic protein (MCP-1)/CCL2, macrophage inflammatory protein (MIP)-1α/CCL3, MIP-1β/CCL4, platelet-derived growth factor-BB (PDGF-BB), regulated upon activation T cell expressed and secreted (RANTES)/CCL5, tumor necrosis factor (TNF), and vascular endothelial growth factor (VEGF). Data acquisition was performed on a Luminex100 analyzer (Luminex Corporation, Austin, Texas, USA) according to the instructions of the manufacturer. Cytokine/Chemokine concentrations were measured in (pg ml^−1^).

### Data Analysis

Patient characteristics were summarized as mean (standard deviation), median (interquartile range; IQR), and counts, as appropriate. Between-group comparisons of biomarker levels across participant groups (Active TB (Group 1), *Mtb* infected (Group 2), recent QFT converters (Group 3), and QFT consistent negatives (Group 4) were carried out applying the Kruskal–Wallis using Dunn’s *post-hoc* test. Within-group comparisons of biomarker levels between baseline and follow-up samples carried out applying Wilcoxon matched paired test.

Analyses aiming to identify diagnostic biosignatures for which a two-step approach was applied (18). In brief, we first applied logistic regression selecting biomarkers for the Lasso regression analysis. A predicted probability >0.5 resulted in classification as participants with *Mtb* infection/disease and <0.5 as participants without *Mtb* infection/disease. The sensitivity and specificity for the identified signatures were defined by their ability to assign a correct high probability (above 0.5) to participants as either TB cases or controls. The diagnostic abilities of the signatures were summarized by means of receiver operator characteristic (ROC) curves (area under the curve [AUC], sensitivity, and specificity). Analyses were carried out using R (R Core Team, 2019) (19) through the interface RStudio (www.rstudio.com).

## Results

### Characterization of the Study Participants at Baseline

A total of 118 study participants were classified into four groups; Active TB (n = 29), *Mtb* infected (n = 30), recent QFT converters (baseline n = 27; follow-up n = 24), and QFT consistent negatives (n = 32). The mean age was 45.6 years (SD± 13.9) for Active TB, 22.0 years (SD± 15.3) for *Mtb* infected, 29.5 years (SD± 19.8) for recent QFT converters, and 18.3 years (SD± 16.9) for QFT consistent negatives. Males constituted 79.3% (23/29) of Active TB, 36.6% (11/30) of *Mtb* infected, 48.1% (13/27) of recent QFT converters, and 50.0% (16/32) of QFT consistent negatives. As expected, the groups with negative QFT at baseline (QFT converters and QFT consistent negatives) all had a TST <10 mm. Therefore, the only meaningful comparison was between Active TB (median TST 15 mm) and *Mtb* infected (median TST 14.5 mm). Interestingly, but consistent with reduced QFT sensitivity in active TB, median IFNγ concentration was higher in *Mtb* infected compared to Active TB. Complete clinical characteristics are shown in **Table 1**.

**Table 1:** Clinical Characteristics of the study groups.

Clinical Characteristics	Active TB (ATB; n = 29)	QFT consistent positives *Mtb* infected (n = 30)	Recent QFT converters baseline samples (n = 27)	Recent QFT converters follow-up samples (n = 24)	QFT consistent negatives (n = 32)
**Demographics**					
Mean age in years (SD)	45.6 ( ± 13.9)	22.0 ( ± 15.3)	29.5 ( ± 19.8)	–	18.3 ( ± 16.9)
Gender (Male)	23	11	13	–	16
**Mycobacterial exposure**					
Known BCG vaccination	11	23	17	–	23
Unknown	3	0	3	–	3
**Tuberculin skin test**					
Positive (>10 mm)	24	30	0	–	0
Median (mm)	15	14.5	8	–	6
**QuantiFERON Gold in tube**					
Positive (≥0.35 IU/ml)	23	30	0	27	0
Test result not available	1^T^	0	0	0	0
Median IFNγ (IU/ml)	3.02	9.7	0.05	0.88	0.02
**Symptoms**					
Cough ≥2 weeks	26	0	0	–	0
Fever ≥1 week	21	0	0	–	0
Weight loss	20	0	0	–	0
**Findings**					
Abnormal Chest X-ray	28	0	0	–	0
HIV test done	12	19	20	–	20
HIV positive	1	0	0	–	0
BMI <18.5 (underweight)	22	14	8	8	12

^^T^^The QTF test was performed, and the test result was not available, but the QTF NIL tube was used for biomarker analysis.

### Cytokines Patterns in Active TB Cases and Exposed Household Contacts at Baseline

**Figure 2:**
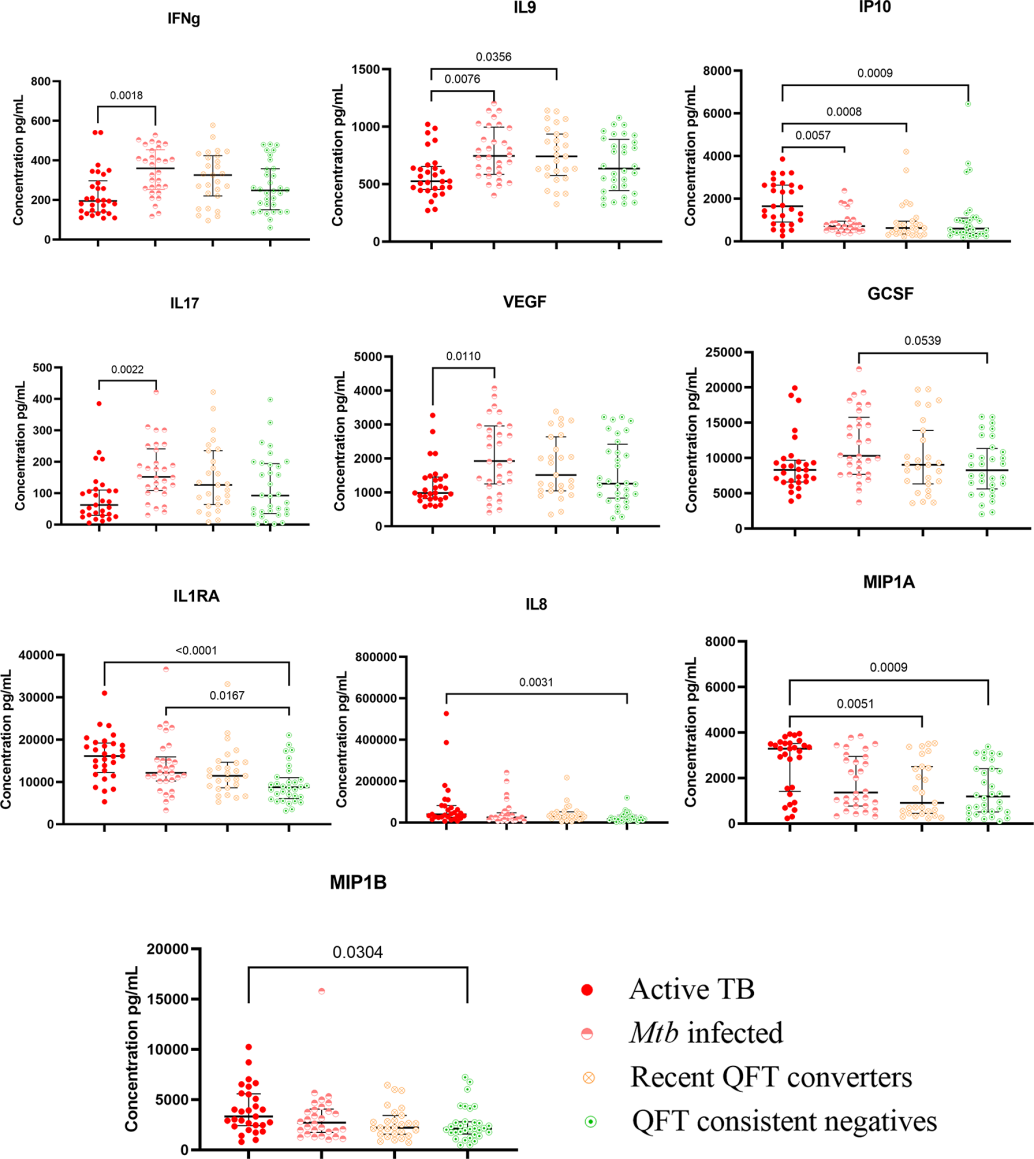
Kruskal–Wallis test with Dunn’s post correction was applied. Scatter-plot graph depicting median cytokine concentrations (pg ml^−1^) from the QFT-GIT supernatants (nil tube) by the Multiplex bio-plex assay. Nil levels (pg ml^−1^) of IFNγ, IL9, IP10, IL-17, VEGF, GCSF, IL1RA, IL8, MIP1α, and MIP1β in patients with active TB (ATB; n = 29), *Mtb* infected (n = 30), recent QFT converters (n = 27), and QFT consistent negatives (n = 32).

Twenty-seven different cytokines, including chemokines and markers of inflammation, were analyzed in QFT-GIT Nil tube supernatants, and compared between Active TB cases and household contacts grouped by *Mtb* infection status following exposure as described. Compared to Active TB, *Mtb* infected exhibited higher levels of the inflammatory T-cell cytokines IFNγ, IL9, and IL17, and the vascular endothelial growth factor (VEGF), whereas the pro-inflammatory IP10, released from innate cells in response to IFNγ was higher in Active TB (**Figure 2**).

Compared to Active TB, QFT consistent negatives had lower levels of the innate pro-inflammatory IL8 promoting neutrophil chemotaxis, macrophage inflammatory protein (MIP)-1α and -1β and IP10, and the anti-inflammatory interleukin 1 receptor antagonist (IL1RA) that inhibits IL-1 effects. Compared to *Mtb* infected, QFT consistent negatives had lower levels of the innate pro-inflammatory cytokine granulocyte colony-stimulating factor (GCSF). QFT consistent negatives also exhibited lower levels of the anti-inflammatory IL1RA compared to Active TB and *Mtb* infected (**Figure 2**).

### Dynamics in Cytokine Patterns in Recent QFT Converters

**Figure 3:**
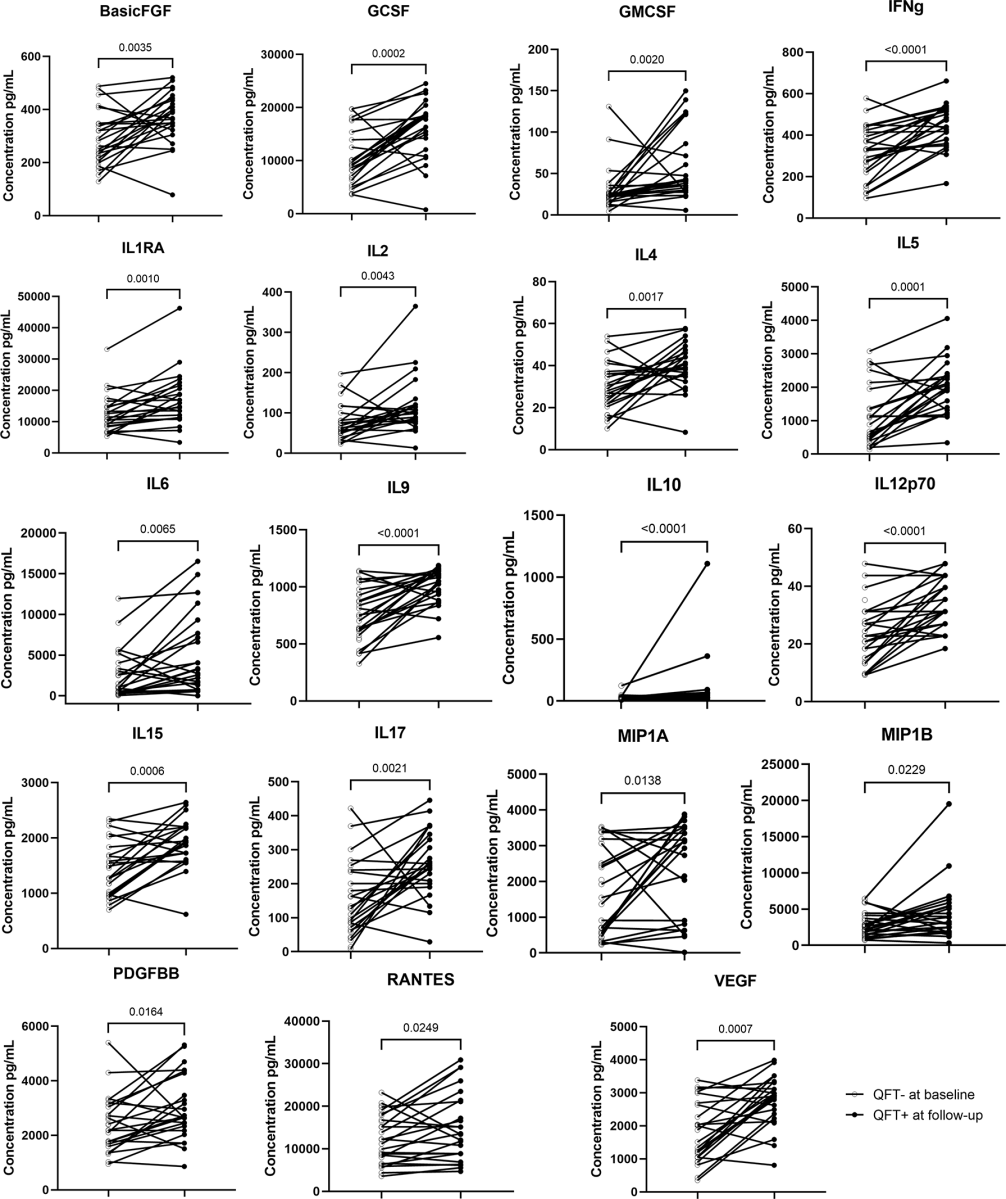
Wilcoxon matched paired test was applied. Symbols and lines graph depicting individual changes in cytokines/chemokines concentrations (pg ml^−1^) from recent QFT converters in baseline samples (n = 27) to recent QFT converters in the follow-up sample (n = 24).

Compared to baseline, the concentrations of 19 out of 27 cytokine/chemokine markers were significantly elevated in recent QFT converters at conversion that occurred within 6 months of continuous exposure to index TB cases (**Figure 3**). All these 19 cytokine/chemokine markers play a role in the development of inflammatory and protective immune responses to microbial invaders by modulating immune cells of both the innate and adaptive immune systems. Notably, all the 19 markers were elevated in the recent QFT converter follow-up samples as compared to the other groups (**Supplementary Table 1**).

### Protein Signatures With a Potential to Discriminate Active TB From *Mtb* Infected and Recent QFT Converters

**Figure 4:**
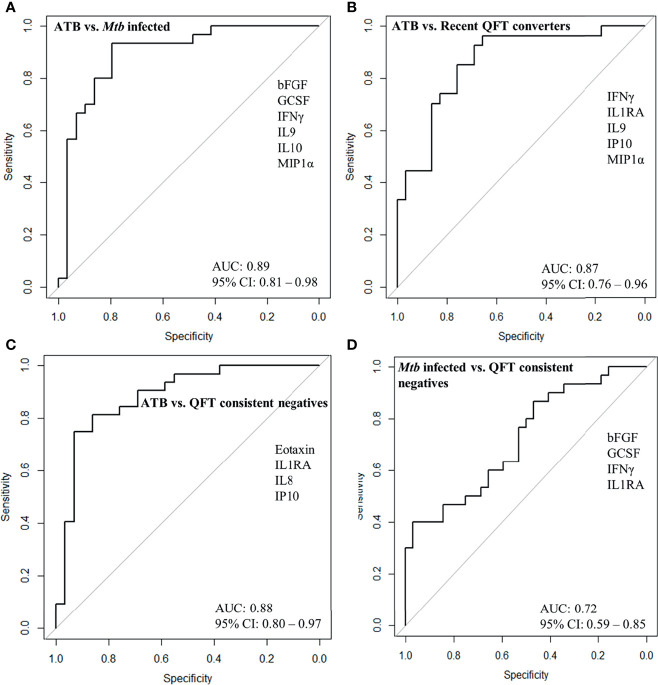
ROC curves for protein signature that distinguishes **(A)** Active TB (ATB; n = 29) from *Mtb* infected (n = 30), **(B)** ATB (n = 29) from recent QFT converters (n = 27), **(C)** ATB (n = 29) from QFT consistent negatives (n = 32), and **(D)** *Mtb* infected (n = 30) from QFT consistent negatives (n = 32).

To identify a protein signature associated with Active TB, univariate feature selection was applied using logistic regression (18), and identified a 6-protein signature, comprising bFGF, GCSF, IFNγ, IL9, IP10, and MIP1α (**Table 2A**) that differentiated Active TB from *Mtb* infected with an AUC of 0.89 (95% CI, 0.81–0.98; **Figure 4A**), sensitivity of 79.3% (95% CI, 60.3–92.0) and specificity of 83.3% (95% CI, 65.3–98.9) (**Table 2B**). Similarly, a 5-protein signature was identified, comprising IFNγ, IL1RA, IL9, IP10, and MIP1α that differentiated Active TB from recent QFT converters with an AUC of 0.87 (95% CI, 0.76–0.96; **Figure 4B**), sensitivity of 75.9% (95% CI, 56.5–89.7) and specificity of 81.5% (95% CI, 61.9–94.4). Interestingly, 4 proteins (IFNγ, IL9, IP10, and MIP1α) overlapped between the two identified signatures, suggesting differential expression of these is associated with active TB disease, rather than infection *per se*.

**Table 2a:** Expression and regression coefficients for each biomarker of the identified protein signature.

ATB vs. *Mtb* infected		ATB vs. Recent QFT converters		ATB vs. QFT consistent negatives		*Mtb* infected vs. QFT consistent negatives	
Cytokines	Slope co-efficient*	Cytokines	Slope co-efficient*	Cytokines	Slope co-efficient*	Cytokines	Slope co-efficient*
bFGF	-7.071	IFNγ	-4.101	Eotaxin	3.472	bFGF	0.868
GCSF	-0.192	IL1RA	0.064	IL1RA	0.173	GCSF	0.073
IFNγ	-0.636	IL9	-1.073	IL8	0.004	IFNγ	0.333
IL9	-1.452	IP10	0.470	IP10	0.331	IL1RA	0.076
IP10	1.021	MIP1α	0.350				
MIP1α	0.640						

*Slope coefficients are scaled-up by a factor of 1000.

**Table 2B:** Identification and performance of protein signature.

	Protein signature	Sensitivity in % (95% CI)	Specificity in % (95% CI)	AUC (95% CI)
**ATB vs. *Mtb* infected**	6-protein signature	79.3 (60.3–92.0)	83.3 (65.3–98.9)	0.89 (0.81–0.98)
**ATB vs. QFT recent converters**	5-protein signature	75.9 (56.5–89.7)	81.5 (61.9–94.4)	0.87 (0.76–0.96)
**ATB vs. QFT consistent negatives**	4-protein signature	86.2 (68.3–96.1)	81.3 (63.6–92.8)	0.88 (0.80–0.97)
***Mtb* infected vs. QFT consistent negatives**	4-protein signature	50.0 (31.3–68.7)	71.9 (53.3–86.3)	0.72 (0.59–0.85)

### Accuracy of Protein Signatures to Assign Therapeutic or Prophylactic TB Treatment

**Figure 5:**
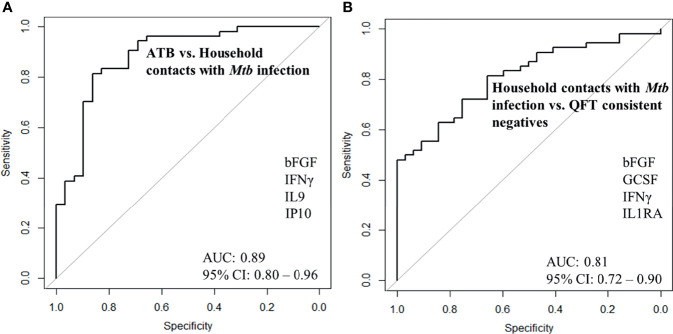
ROC curves for signature that distinguishes **(A)** Active TB (n = 29) from Household contacts with *Mtb* infection (n = 30), **(B)** Household contacts with *Mtb* infection (n = 30) from QFT consistent negatives (n = 32).

Next, we merged household contacts that could profit from preventive TB treatment, namely *Mtb* infected, sampled at baseline, and recent QFT converters, sampled at the time of/after QFT conversion. This group, called Household contacts with *Mtb* infection (merged *Mtb* infected + recent QFT converters), was then compared against Active TB to identify a protein signature. Univariate feature selection was followed by lasso regression analysis. A 4-protein signature was identified, comprising bFGF, IFNγ, IL9, and IP10 (**Table 3A**) that differentiated Active TB from Household contacts with *Mtb* infection with an AUC of 0.89 (95% CI, 0.80–0.96; **Figure 5A**), sensitivity of 72.4% (95% CI, 52.8–87.3) and specificity of 92.6% (95% CI, 82.1–97.9) (**Table 3B**).

**Table 3a:** Expression and regression coefficients for each biomarker of the identified protein signature.

ATB vs. Household contacts with *Mtb* infection		Household contacts with *Mtb* infection vs. QFT consistent negatives	
Cytokines	Slope co-efficient*	Cytokines	Slope co-efficient*
bFGF	-3.92	bFGF	1.182
IFNγ	-2.37	GCSF	0.078
IL9	-1.90	IFNγ	1.978
IP10	0.401	IL1RA	0.074

*Slope coefficients are scaled-up by a factor of 1000.

**Table 3B:** Identification and performance of protein signature.

	Protein signature	Sensitivity in % (95% CI)	Specificity in % (95% CI)	AUC (95% CI)
**ATB vs. Household contacts with *Mtb* infection**	4-protein signature	72.4 (52.8–87.3)	92.6 (82.1–97.9)	0.89 (0.80–0.96)
**Household contacts with *Mtb* infection vs. QFT consistent negatives**	4-protein signature	87.0 (75.1–94.6)	62.5 (43.7–78.9)	0.81 (0.72–0.90)

### Protein Signatures Distinguishing QFT Consistent Negatives From Active TB and *Mtb* Infected

QFT consistent negatives were satisfactorily discriminated from Active TB by a 4-protein signature, comprising Eotaxin, IL1RA, IL8, and IP10 that differentiated with an AUC of 0.88 (95% CI, 0.80–0.97; **Figure 4C**), sensitivity of 86.2% (95% CI, 68.3–96.1), and specificity of 81.3% (95% CI, 63.6–92.8). In addition, QFT consistent negatives could to some extent be discriminated from *Mtb* infected by another 4-protein signature comprising bFGF, GCSF, IFNγ, and IL1RA (**Table 2A**) with an AUC of 0.72 (95% CI, 0.59–0.85; **Figure 4D**), sensitivity of 50.0% (95% CI, 31.3–68.7), and specificity of 71.9% (95% CI, 53.3–86.3) (**Table 2B**). Notably, IL1RA overlapped between the two identified signatures.

### Accuracy of Protein Signatures to Safely Assign no TB Treatment

Next, Household contacts with *Mtb* infection (merged *Mtb* infected + recent QFT converters) were compared to QFT consistent negatives by lasso analysis. A 4-protein signature was identified, comprising bFGF, GCSF, IFNγ, and IL1RA (**Table 3A**) that differentiated Household contacts with *Mtb* infection from QFT consistent negatives with an AUC of 0.81 (95% CI, 0.72–0.90; **Figure 5B**), sensitivity of 87.0% (95% CI, 75.1–94.6), and specificity of 62.5% (95% CI, 43.7–78.9; **Table 3B**) for safe assignment to no TB treatment.

## Discussion

The natural history of TB is characterized by a dynamic spectrum of disease, in which newly infected individuals are at the highest risk of developing active disease, but may also enter a state of long-term latent infection from which they may progress to disease many years after infection (20). Understanding where infected individuals are in this spectrum from infection to disease progression is the key to appropriate management and treatment. The purpose of contact investigations following identification of a TB index case is early case detection to limit morbidity and further transmission. This can be achieved by i) identification of concomitant TB cases in need of a therapeutic course of TB treatment, and ii) identification of individuals with recently acquired *Mtb* infection and subsequent elevated risk of TB progression who would benefit from preventive therapy or attentive clinical surveillance (21). We identified a 6-protein signature (bFGF, GCSF, IFNγ, IL9, IP10, and MIP1α) that distinguished Active TB from *Mtb* infected, and a 4-protein signature (Eotaxin, IL1RA, IL8, and IP10) that distinguished Active TB from QFT consistent negatives with AUCs of 0.89 and 0.88, respectively.

In order to guide the assignment of HHCs to full-length TB treatment (Active TB) or preventive TB treatment (*Mtb* infected), we compared Active TB vs. Household contacts with *Mtb* infection (merged *Mtb* infected + recent QFT converters) and identified a 4-protein signature (bFGF, IFNγ, IL9, and IP10) with a specificity of 92.6%. Even with an accurate biomarker signature to identify concomitant TB cases in a household, we acknowledge the need for respiratory samples and microbiological verification before initiation of TB treatment to ascertain the diagnosis and drug sensitivity. Nevertheless, a screening test for *Mtb* infection could accurately rule out concomitant TB and limit the number of HHCs in need for the sampling for a respiratory specimen and positively assigning HHCs for preventive TB treatment with low risk of undertreating concomitant incipient and subclinical TB cases. Hopefully, the screening test would also identify and secure appropriate treatment of extrapulmonary TB cases for which microbiological verification is far more challenging. Further, to identify HHCs for whom treatment could safely be withheld due to likely natural TB immunity, we compared QFT consistent negatives with Household contacts with *Mtb* infection and identified a 4-protein signature (bFGF, GCSF, IFNγ, and IL1RA) that correctly classified QFT consistent negatives with an AUC of 0.81 and a disappointing specificity of 62.5%. Interestingly, bFGF and IFNγ overlapped between the two identified signatures. In TB pathology, T cells produce IFNγ, which aids in protective immunity against *M. tuberculosis* by activating macrophages, allowing them to eliminate *Mtb* more effectively (22). Still, although essential for anti-mycobacterial responses, IFNγ alone is not sufficient, and high levels of IFNγ-producing T cells do not correlate with immune protection (23). bFGF is also associated with tissue regeneration and angiogenesis, but we observed reduced levels in Active TB compared to HHCs with *Mtb* infection (QFT consistent positives).

The innate immune response that controls *Mtb* infection starts with pathogen recognition and uptake by resident tolerant alveolar macrophages in which *Mtb* finds a niche for replication. Once the bacterial load reaches beyond “the tolerant threshold”, local early innate responses are activated and start producing cytokines, chemokines, and antimicrobial molecules resulting in bacterial killing/restricted growth and initiation of adaptive immune responses (24). The relevance of exploring the biomarker potential of cytokines and chemokines, and the importance of the IFNγ cytokine in TB immunology has already been mentioned. Regarding chemokines, these are essential for the recruitment of the first line of innate immune effector cells to infection and inflammatory sites and the deployment of natural immune sentinels at mucosal barriers (25). Further, the plasma levels of chemokines have the capacity to influence host immunity to sustain the protective immune response (26). Another likely advantage is that many chemokines, namely, MCP-1, MIP1α, MIP-1β, and IP-10, in adults have been reported probably not to associate with malnutrition, a major risk factor for TB globally (27), whereas this is more uncertain with TST and QFT results. Studies have reported that IP10 is a potential biomarker for TB and contributes to restricting *Mtb* replication in host tissues (28–32). Interestingly, mRNA levels of the cytokines *CCL4* (MIP1β), *CXCL10* (IP10) have been reported to decrease following isoniazid preventive treatment (33), suggesting a broad potential for chemokine biomarker tools in diagnosis and prediction of progression and evaluation of treatment response (34–36).

In our study, QFT conversion was indeed reflected in alterations in the analyzed soluble markers as nearly two-thirds (19/27) had increased median concentration from baseline to the time of/after QFT conversion (recent QFT converters). This provides insight in the early chemokines/cytokines-driven inflammatory response induced by *Mtb* infected/activated macrophages that result in recruitment and activation of innate and adaptive immune cells to the lung (26, 37). In response to granulocyte–macrophage colony-stimulating factor, monocytes, neutrophils, epithelial cells, and adipocytes produce IL-1RA (38), the level of IL1RA was elevated in Active TB in this study. A study has reported that the level of IL-1RA was higher in patients with delayed treatment response than those with a favorable response to therapy (39). IL-9 is mainly produced by Th2 cells, promotes the pleural mesothelial cell repairing, and inhibits IFNγ-induced pleural mesothelial cell apoptosis (33). This is also consistent with a host protective response that aims to prevent excessive pathology (40). IL-9 is involved in the immune-pathogenesis of inflammatory diseases and maintaining immune tolerance, putting it in a similar role to the IL1RA. In TB, IL-17 has been suggested to protect against disease progression and pathological damage in the lung by initial neutrophil recruitment after *Mtb* infection (41). As reported in a previous study (42), the levels of IL-9 and IL-17 (measured in QFT-Nil tube plasma samples) were reduced in active TB compared to *Mtb* infection. We report that the pro-inflammatory IP10 was higher in Active TB compared to the other groups, and this finding is consistent with previous studies conducted in various populations (34, 42, 43). IP-10 also plays a chemotactic role, though it seems to be more critical for T cells. The elevated expression of these cytokines in active TB is associated with the infiltration and consolidation seen in the lungs of TB patients and suggests the elevated expression of these genes acts as a marker for TB pathology rather than *Mtb* infection (38). In the present study, the levels of chemokines IL-8, MIP-1α and MIP-1β and were elevated in active TB, IL8 and MIP-1α attract both lymphocytes and polymorphonuclear neutrophils. IL-8 is involved in the recruitment of leukocytes and the formation of granulomas. Further, MIP-1α from neutrophils may contribute to the recruitment of mononuclear cells after neutrophils have accumulated. Therefore, the production of IL-8 and MIP-1α may be greater in suppurative inflammation than in mycobacterial inflammation (39, 44).

The study reported here has some limitations, principally the fact that it analyzed a limited number of cytokines and chemokines. Moreover, the household contacts were younger than the TB index cases and there was an uneven sex distribution. Immune responses changes with age and this may have had an impact on host protein levels and the identified protein signatures. Further, the risk of TB-exposure *per se*, but also for repeated TB exposure, increases with age. By design, eligible TB index cases were adults, thereby inducing an inevitable age bias in the Active TB group. A sub-analysis undertaken in the QFT consistent negatives (n = 32), whom can be assumed to be unaffected by the probable age-dependant increase in TB exposure, did not find any significant correlation between age and cytokine concentrations. In addition, no formal sample size calculation was carried out since the maximum sample size was limited by the availability of samples for biomarker analysis and the inability to cross-validate the identified proteomic signature due to the lack of comparable samples from other cohorts. In this study, TB treatment was started in index TB cases based on recommendations, which included the presence of clinical symptoms and also microbiological and/or radiological findings. Even with a valid biomarker signature to identify concomitant TB cases in a household, we acknowledge the need for respiratory samples and microbiological verification before initiation of TB treatment to ascertain the diagnosis and drug sensitivity. Nevertheless, based on these results, further exploration of biosignatures and validation of suggested signatures in broad settings, is warranted in order to establish their potential and eventual use to guide optimal TB case detection and assignment to therapeutic and preventive treatment to *Mtb* infected individuals. Optimal individual management is currently our best option to change TB epidemiology.

## Data Availability Statement

The original contributions presented in the study are included in the article/**Supplementary Material**. Further inquiries can be directed to the corresponding authors.

## Ethics Statement

Ethical approval for this study was obtained from the Institutional Ethical Review Board (IERB) of St. John’s Medical College, Bangalore (IERB/1/527/08). The material transfer agreement between St. John’s Medical College, Bangalore, and the University of Bergen, Norway, was obtained from the Department of Biotechnology, Government of India (No. BT/Med.II/Adv (SS)/Misc./02/2012). Ethical approval was also obtained (Ref no: 2018/1614 D) from Western Norway’s Regional Committee for Medical and Health Research Ethics. Written informed consent to participate in this study was provided by the participants’ legal guardian/next of kin.

## Author Contributions

DS, SJ, TMD, CR, and HMSG conceptualized and designed the biomarker study. MV coordinated patient recruitment and follow-up. DS and SJ wrote the manuscript with contributions from TMD, CR, and HMSG. DS performed all laboratory experiments, data analysis and generated the tables and figures. CR supervised the statistical analysis, wrote the section on statistical analysis, and reviewed the manuscript. HMSG had primary responsibility for the final content of the manuscript. All authors listed have made a substantial, direct, and intellectual contribution to the work and approved it for publication.

## Funding

The study is supported by the Research Council of Norway Global Health and Vaccination Research (GLOBVAC) projects: RCN 179342, 192534, and 248042, the University of Bergen (Norway); the St. John’s Research Institute, Bangalore. Helse-vest grant (F-10441).

## Conflict of Interest

TMD is an employee of and holds shares in the GSK group of companies but participated in the current work as an independent investigator.

The remaining authors declare that the research was conducted in the absence of any commercial or financial relationships that could be construed as a potential conflict of interest.

## Publisher’s Note

All claims expressed in this article are solely those of the authors and do not necessarily represent those of their affiliated organizations, or those of the publisher, the editors and the reviewers. Any product that may be evaluated in this article, or claim that may be made by its manufacturer, is not guaranteed or endorsed by the publisher.
